# Supplementary material for: Splice-Junction-Based Mapping of Alternative Isoforms in the Human Proteome
Source: Cell Rep. Author manuscript; Available in PMC 2020 Jan 15. (PMC6961840; doi:10.1016/j.celrep.2019.11.026)
Supplement: 3 [file NIHMS1546469-supplement-3.zip › DF2/PXD006675/InferiorVenaCava-13-Q14643-HLATGHYLAAEVDPDQDASR.pdf]

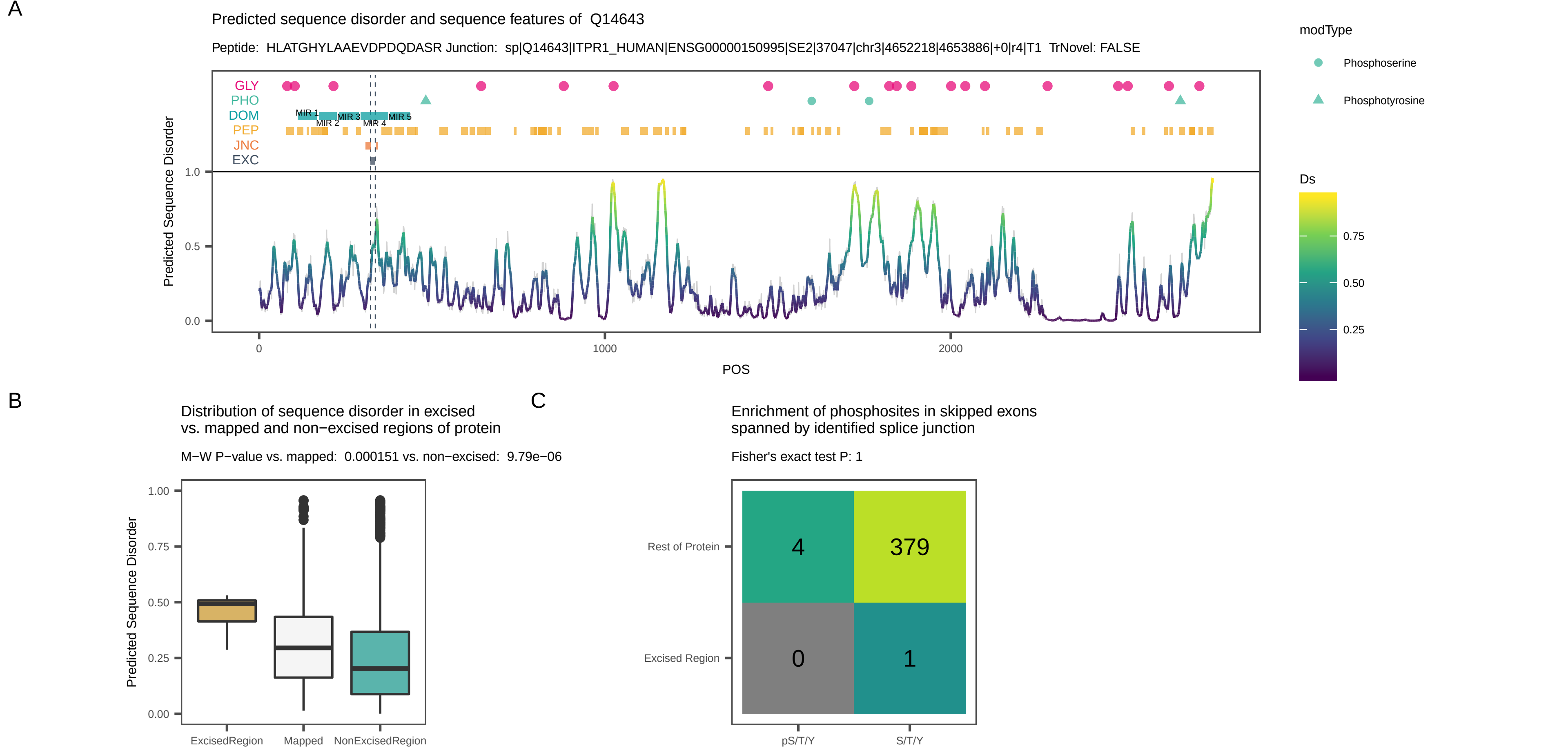
A
Predicted sequence disorder and sequence features of Q14643
Peptide: HLATGHYLAAEVDPDQDASR Junction: sp|Q14643|ITPR1_HUMAN|ENSG00000150995|SE2|37047|chr3|4652218|4653886|+0|r4|T1 TrNovel: FALSE
GLY
PHO
DOM
PEP
JNC
EXC
MIR 1
MIR 2
MIR 3
MIR 4
MIR 5
Predicted Sequence Disorder
1.0
0.5
0.0
0
1000
2000
POS
modType
Phosphoserine
Phosphotyrosine
Ds
0.75
0.50
0.25
B
Distribution of sequence disorder in excised vs. mapped and non−excised regions of protein
M−W P−value vs. mapped: 0.000151 vs. non−excised: 9.79e−06
Predicted Sequence Disorder
1.00
0.75
0.50
0.25
0.00
ExcisedRegion
Mapped
NonExcisedRegion
C
Enrichment of phosphosites in skipped exons spanned by identified splice junction
Fisher's exact test P: 1
Rest of Protein
Excised Region
4
379
0
1
pS/T/Y
S/T/Y
